# Statin Therapy Is Associated with Reduced Risk of Peptic Ulcer Disease in the Taiwanese Population

**DOI:** 10.3389/fphar.2017.00210

**Published:** 2017-04-28

**Authors:** Chun-Jung Lin, Wei-Chih Liao, Yu-An Chen, Hwai-Jeng Lin, Chun-Lung Feng, Cheng-Li Lin, Ying-Ju Lin, Min-Chuan Kao, Mei-Zi Huang, Chih-Ho Lai, Chia-Hung Kao

**Affiliations:** ^1^Department of Urology, University of Texas Southwestern Medical CenterDallas, TX, USA; ^2^Graduate Institute of Clinical Medical Science, China Medical UniversityTaichung, Taiwan; ^3^Department of Pulmonary and Critical Care Medicine, China Medical University HospitalTaichung, Taiwan; ^4^School of Medicine, Graduate Institute of Basic Medical Science, China Medical UniversityTaichung, Taiwan; ^5^Division of Gastroenterology and Hepatology, Department of Internal Medicine, School of Medicine, College of Medicine, Taipei Medical UniversityTaipei, Taiwan; ^6^Division of Gastroenterology and Hepatology, Department of Internal Medicine, Shuang-Ho HospitalNew Taipei, Taiwan; ^7^Department of Internal Medicine, China Medical University HospitalTaichung, Taiwan; ^8^Management Office for Health Data, China Medical University HospitalTaichung, Taiwan; ^9^College of Medicine, China Medical UniversityTaichung, Taiwan; ^10^Genetic Center, Department of Medical Research, School of Chinese Medicine, China Medical University and HospitalTaichung, Taiwan; ^11^Department of Microbiology and Immunology, Graduate Institute of Biomedical Sciences, Chang Gung UniversityTaoyuan, Taiwan; ^12^Department of Nursing, Asia UniversityTaichung, Taiwan; ^13^Department of Pediatrics, Molecular Infectious Disease Research Center, Chang Gung Children's Hospital and Chang Gung Memorial HospitalTaoyuan, Taiwan; ^14^Department of Bioinformatics and Medical Engineering, Asia UniversityTaichung, Taiwan; ^15^Department of Nuclear Medicine, PET Center, China Medical University HospitalTaichung, Taiwan

**Keywords:** case-control study, cholesterol, peptic ulcer disease, statin

## Abstract

Although statin use may affect the severity of chronic gastritis and gastric cancer, no data exists about the relationship between statin therapy and risk of peptic ulcer disease (PUD) in patients. We investigated the effect of statin use and the incidence of PUD from the Taiwan National Health Insurance Research Database (NHIRD). A total of 35,194 patients records for medical claims were enrolled. We performed a population-based case-control analysis to compare the incidence of PUD in patients who were prescribed statins and that in patients who were not. In the univariate logistic analysis, we found that statin was not significant risk of PUD. However, a multivariate model indicates that satin use was significantly associated with a reduced risk of PUD (adjusted odds ratio [aOR] = 0.87, 95% CI = 0.82–0.93, *P* < 0.001). The cumulative defined daily dose (DDD) was analyzed. Patients who prescribed fluvastatin ≥280 DDD, atorvastatin ≥200 DDD, and pravastatin ≥130 DDD dramatically decreased risk for PUD (aOR = 0.58, 0.67, and 0.71; 95% CI = 0.46–0.74, 0.57–0.78, and 0.56–0.91, respectively). Our results showed that statin therapy reduced the risk of PUD and this was associated with the high cumulative DDD of prescribed statins. This study reveals that active use of statins to be associated with decreased risk for PUD.

## Introduction

Peptic ulcer disease (PUD) has been known can be caused by *Helicobacter pylori* infection and widespread use of non-steroidal anti-inflammatory drugs (NSAIDs; Malfertheiner et al., 2009). Despite the cases of *H. pylori* have been decreasing in eradication treatment over the last decade, the incidence of PUD is still at high levels in some countries. Without treatment, the complications to PUD may develop into bleeding, perforation, and obstruction which lead to increase hospitalizations and mortalities (Lau et al., 2011).

Statins are inhibitors for 3-hydroxy-3-methyl-glutaryl-coenzyme A (HMG-CoA) reductase and have been found to play a protective role in several bacteria-associated diseases (Jerwood and Cohen, 2008; Nseir et al., 2013; Skerry et al., 2014). Previous studies have reported that combination treatments, including triple therapies (consisting of a proton-pump inhibitor, amoxicillin, and clarithromycin) prescribed along with statins, accelerate *H. pylori* clearance and ameliorate ulcer development (Tariq et al., 2007; Yamato et al., 2007; Nseir et al., 2012).

Statin use reduces cardiovascular disease-related morbidity and mortality in patients with and without coronary diseases (Maron et al., 2000). In addition to cholesterol lowering effects, statins have anti-inflammatory properties including modulation of immune responses, regulation of MHC expression, mucosal proliferation, and secretory activity (Kwak et al., 2000; Weitz-Schmidt et al., 2001). The additional benefits of statins included gastroprotective effects and attenuation of peptic ulcer development (Tariq et al., 2007; Heeba et al., 2009). However, the clinical relevance of statins on gastrointestinal disorders require further investigation.

It has been reported that statin use may decrease the incidence of chronic gastritis, and reduce the risk of several types of cancers (Nseir et al., 2010; Chiu et al., 2011; Singh and Singh, 2013; Singh et al., 2013; Liu et al., 2015; Ananthakrishnan et al., 2016). In addition, our recent study by combining a cell-based study with a nationwide population analysis revealed that statin use attenuated the risk for *H. pylori*-associated gastric cancer (Lin et al., 2016). However, previous studies have suggested that statin use was not associated with decrease the risk of peptic ulcer or reflux esophagitis (Fujii et al., 2009). An animal study also indicated that a statin was ineffective for suppressing gastritis and chemoprevention of gastric carcinogenesis in gerbils (Toyoda et al., 2009). The reports are clearly controversial, but there has been no large-scale epidemiologic research on the inhibitory effects of statins for treating PUD. Therefore, it is worth investigating whether statin use mitigated PUD in patients by analyzing a nationwide database. In the present study, we performed a nationwide population-based case-control analysis to compare the incidence of PUD patients who were prescribed statins and that in patients who were not. Our results indicate that statins might alleviate the risk of PUD.

## Materials and methods

### Data source

The National Health Insurance (NHI) program was established in Taiwan in 1995 and has enrolled ≤99% of the Taiwanese population (Cheng, 2003). It is contracted with 97% of the medical providers nationwide (Cheng et al., 2011). The National Health Research Institutes (NHRI) is responsible for managing the insurance claims data reported to the Bureau of Health Insurance. For research purposes, the NHRI compiles all medical claims in the NHI program and releases the database annually to the public. This case-control study used data from Taiwan's NHI electronic records system, which contains all medical claims from 1996 to 2010. The National Health Insurance Research Database (NHIRD) contains medical information, including data on inpatient and outpatient care facilities, drug prescriptions, insurant sex and date of birth, date of visit or hospitalization, and diagnoses coded in the format of the International Classification of Disease, 9th Revision, Clinical Modification (ICD-9-CM) that has been described in our previous studies (Chen et al., 2015; Hsu C. C. et al., 2015; Hsu Y. C. et al., 2015). Patient consent is not required to access the NHIRD. This study has approved by the Institutional Review Board of China Medical University in Central Taiwan (CMUH104-REC2-115).

### Study patients

**Figure 1:**
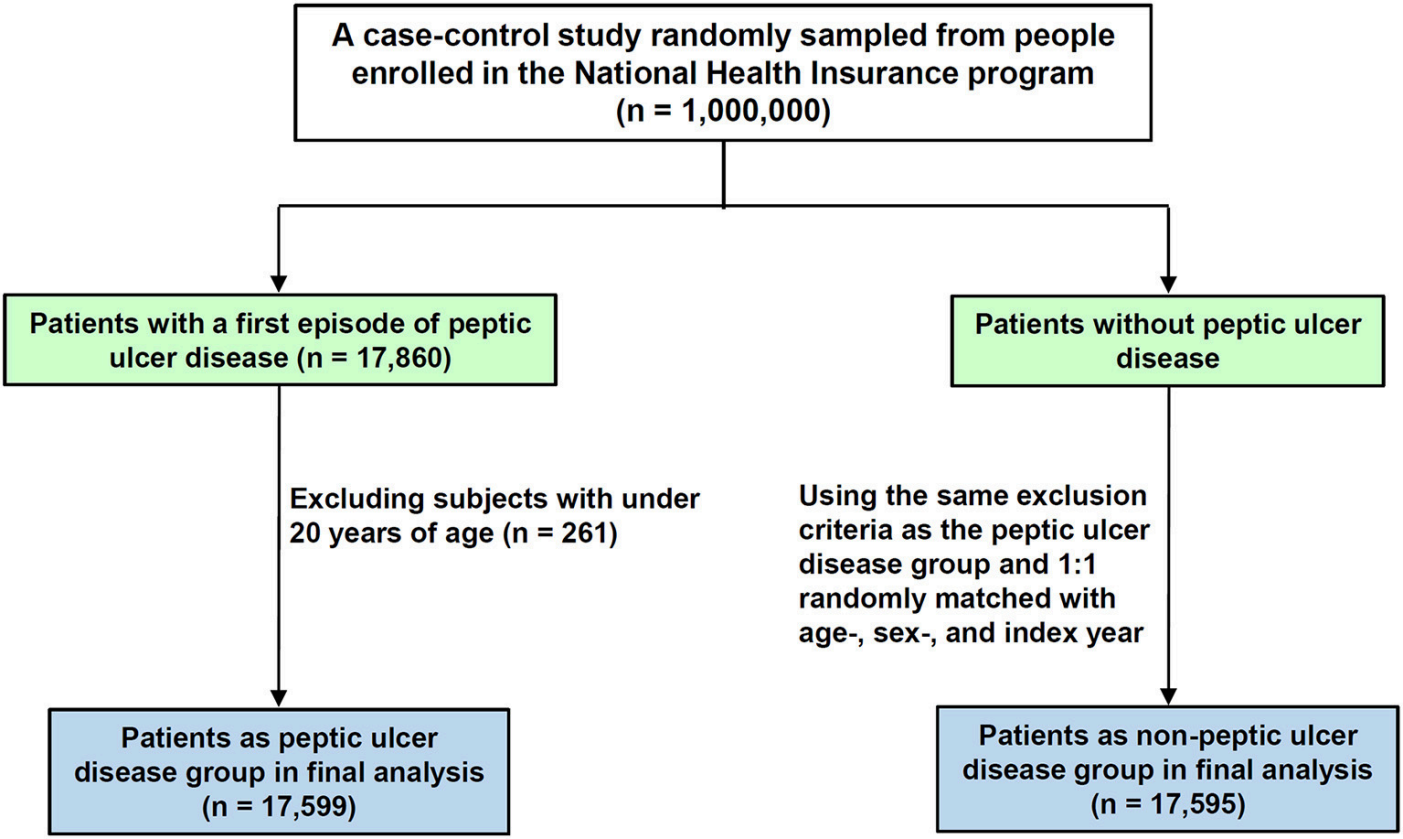
**Flowchart of the patient identification and selection**.

The case group included patients with hyperlipidemia (ICD-9-CM 272) who were newly diagnosed with PUD (ICD-9-CM 531-535) from 2005 to 2010. Figure 1 shows the flowchart for selecting the study groups. Patients aged <20 years were excluded. The date of the first diagnosis of PUD was used as the index date. For each PUD patient, one hyperlipidemia patient without PUD from the same period was selected using the same exclusion criteria, frequency-matched for sex and age (in 5-y groups). Overall, 17,599 patients with PUD and 17,595 patients without PUD were included.

### Stratification of urbanization level

The NHRI is the institute responsible for managing the insurance claims data for Taiwan. The NHRI stratified Taiwan into 7 urbanization levels, based not only on population density (people/km^2^) but also on the proportion of residents attaining higher education, the number of elderly citizens, the degree of agricultural activity, and the number of physicians per 100,000 people in each area. In this study, Level 1 represents areas with the highest population density and socioeconomic status, and Level 7 represents the lowest. Since very few people lived in the more rural areas of Levels 4–7, we grouped these 4 areas into Level “4.”

### Comorbidities, medication, and measurement of statin prescription

Major comorbidities considered as covariates were tobacco dependency (ICD-9-CM code 305.1), chronic obstructive pulmonary disease (COPD) (ICD-9-CM codes 491, 492, 496), diabetes (ICD-9-CM code 250), stroke (ICD-9-CM codes 430–438), cirrhosis (ICD-9-CM code 571), coronary artery disease (CAD) (ICD-9-CM codes 410–414), hypertension (ICD-9-CM codes 401–405), gastroesophageal reflux disease (ICD-9-CM code 530.81 and 530.11), gastric polyp (ICD-9-CM code 211.1), and *H. pylori* infection (ICD-9-CM code 041.86) at the baseline. The potential medications for PUD included aspirin, and non-steroidal anti-inflammatory drugs (NSAIDs).

Statin usage records were retrieved from the ambulatory and inpatient claims data. According to the total supply in days and the quantity of statin, the cumulative defined daily dose (DDD) of each type of statin, including simvastatin (ATC C10AA01), lovastatin (ATC C10AA02), pravastatin (ATC C10AA03), fluvastatin (ATC C10AA04), atorvastatin (ATC C10AA05), and rosuvastatin (ATC C10AA07), was calculated. For each type of statin, the cumulative DDD was divided into two levels according to the median dose.

## Statistical analysis

For NHI database analysis, the baseline characteristics of the PUD and non-PUD groups were compared using a chi-square test. Crude and adjusted odds ratio (aOR) and 95% confidence intervals (CI) for factors associated with the risk of PUD were estimated using univariable and multivariable logistic regression models. All statistical analyses were performed using SAS statistical software for Windows (Version 9.3; SAS Institute, Inc, Cary, NC, USA). Results from experimental data analyses are expressed as mean ± SEM. Student's *t*-tests were used to determine the statistical significance of differences in experimental results between the two groups. A *P* < 0.05 was considered significant.

## Results

### Demographic characteristics of patients

In this study, we first evaluated 35,194 patients with hyperlipidemia, aged ≥20 years (Figure 1). Of the patients with PUD, 51.2% were men and 60.2% were aged 40–64 years (Table 1). The mean age of the PUD and non-PUD patients was 53.2 (±14.3) and 53.1 (±14.3) years, respectively. Differences between the two groups with respect to age (*P* = 0.34), sex (*P* = 0.99), and use of statins (*P* = 0.40) were not significant. Aspirin and NSAIDs medications were more prevalent in the PUD group at the baseline (*P* = 0.001) compared with the non-PUD group. In addition, most patients of both groups tended to reside in urbanized areas higher than level 2 (61.2 vs. 65.6%) and had monthly income level between 15,000 and 19,999 New Taiwan Dollars (48.0 vs. 43.4%). Patients with PUD were more likely to have baseline comorbidities than those who did not have PUD.

**Table 1:** **Baseline characteristics of PUD and non-PUD groups^**a**^**.

	**PUD**				
	**No (*****n*** = **17595)**		**Yes (*****n*** = **17599)**		
	***n***	**%**	***n***	**%**	***P*-value^b^**
Age group (year)					0.99
20–39	3,194	18.2	3,193	18.1	
40–64	10,586	60.2	10,587	60.2	
65–74	2,524	14.3	2,524	14.3	
≥75	1,291	7.34	1,295	7.36	
Mean ± SD (year)	53.1 ± 14.3		53.2 ± 14.3		0.34
Gender					0.99
Female	8,579	48.8	8,581	48.8	
Male	9,016	51.2	9,018	51.2	
Urbanization level^c^					<0.001
1 (highest)	6,382	36.3	5,551	31.5	
2	5,163	29.3	5,224	29.7	
3	2,790	15.9	3,021	17.2	
4 (lowest)	3,260	18.5	3,803	21.6	
Monthly Income (NT$)^d^					<0.001
<15,000	3,684	20.9	3,556	20.2	
15,000–19,999	7,643	43.4	8,455	48.0	
≥20,000	6,268	35.6	5,588	31.8	
Medications					
Statins	3,670	20.9	3,735	21.2	0.40
Aspirin	2,553	14.5	3,003	17.1	0.001
NSAIDs	8,821	50.1	11,394	64.8	0.001
Baseline comorbidities					
Tobacco dependency	228	1.30	351	1.99	<0.001
COPD	1,132	6.43	1,876	10.7	<0.001
Diabetes	3,553	20.2	3,742	21.3	0.01
Stroke	1,528	8.69	1,787	10.2	<0.0001
Cirrhosis	3,927	22.3	5,147	29.3	<0.0001
CAD	2,318	13.2	3,196	18.2	<0.0001
Hypertension	6,930	39.4	7,531	42.8	<0.0001
Gastroesophageal reflux disease	96	0.55	779	4.43	<0.0001
Gastric polyp^e^	5	0.03	25	0.14	<0.0001

a *CAD, coronary artery disease; COPD, chronic obstructive pulmonary disease; PUD, peptic ulcer diseases*.

b *Chi-square test and Student's t-test were used to compare the PUD and non-PUD groups*.

c *The urbanization level was categorized by the population density of the residential area into four levels, with level 1 as the most urbanized and level 4 as the least urbanized*.

d *New Taiwan (NT) Dollars per month. One NT Dollar equals to 0.03 US Dollar*.

e *Student's t-test along with Fisher exact were used to compare the PUD and non-PUD groups. Data are presented as the number of patients in each group and percentages*.

### Prescribed statins reduce the risk of PUD

We then analyzed the crude OR and adjusted OR (aOR) of PUD risk according to urbanization level, monthly income, statin use, and comorbidities. As shown in Table 2, the low monthly income had an aOR of 1.09 for PUD compared with those of higher monthly income (95% CI = 1.03–1.16). The aOR of PUD was 1.26 (95% CI = 1.19–1.35) for the living in lowest urbanization level, compared with the living in highest urbanization level. A univariate logistic analysis identified 11 comorbidities as risk factors for PUD (all *P* < 0.05; Table 1). However, the multivariate model indicates that tobacco dependency, COPD, cirrhosis, CAD, gastroesophageal reflux disease, gastric polyps, and *H. pylori* infection were all significantly associated with an increased risk of PUD (Table 2). After adjusting for age, sex, urbanization level, monthly income, aspirin, NSAIDs and comorbidities, PUD risk was lower in statin users when compared with non-users (aOR = 0.87, 95% CI = 0.82–0.93, *P* < 0.001).

**Table 2:** **Odds ratios and 95% confidence intervals of PUD associated with statin use and covariates^**a**^**.

	**Crude**		**Adjusted^b^**	
**Variable**	**OR**	**(95% CI)**	**OR**	**(95% CI)**
**URBANIZATION LEVEL^c^**				
1 (highest)	1	(Reference)	1	(Reference)
2	1.16	(1.10, 1.23)^***^	1.14	(1.08, 1.21)^***^
3	1.25	(1.17, 1.33)^***^	1.24	(1.16, 1.32)^***^
4 (lowest)	1.34	(1.26, 1.42)^***^	1.26	(1.19, 1.35)^***^
**MONTHLY INCOME (NT$)^d^**				
<15,000	1.08	(1.02, 1.15)^**^	1.09	(1.03, 1.16)^*^
15,000–19,999	1.24	(1.18, 1.30)^***^	1.21	(1.15, 1.27)^***^
≥ 20,000	1	(Reference)	1	(Reference)
**MEDICATIONS**				
Statins	1.02	(0.97, 1.08)	0.87	(0.82, 0.93)^***^
Aspirin	1.21	(1.14, 1.28)^***^	0.96	(0.89, 1.03)
NSAIDs	1.83	(1.75, 1.91)^***^	1.74	(1.66, 1.82)^***^
**BASELINE COMORBIDITIES**				
Tobacco dependency	1.55	(1.31, 1.83)^***^	1.43	(1.21, 1.70)^***^
COPD	1.74	(1.61, 1.87)^***^	1.48	(1.37, 1.61)^***^
Diabetes	1.07	(1.01, 1.12)^*^	0.98	(0.92, 1.04)
Stroke	1.19	(1.11, 1.28)^***^	1.02	(0.94, 1.10)
Cirrhosis	1.44	(1.37, 1.51)^***^	1.38	(1.31, 1.45)^***^
CAD	1.46	(1.38, 1.55)^***^	1.34	(1.25, 1.44)^***^
Hypertension	1.15	(1.10, 1.20)^***^	0.99	(0.94, 1.04)
Gastroesophageal reflux disease	8.44	(6.82, 10.4)^***^	8.20	(6.61, 10.2)^***^
Gastric polyp	5.00	(1.92, 13.1)^**^	4.16	(1.56, 11.1)^**^
*H. pylori* infection	75.6	(10.6, 599.2)^***^	78.9	(11.1, 562.6)^***^

a *CAD, coronary artery disease; CI, confidence intervals; COPD, chronic obstructive pulmonary disease; OR, odds ratios; PUD, peptic ulcer diseases*.

b *Adjusted for age, sex, urbanization level, monthly income, aspirin, NSAIDs, tobacco dependency, COPD, diabetes, stroke, cirrhosis, CAD, hypertension, gastroesophageal reflux disease, gastric polyp, and H. pylori infection*.

c *The urbanization level was categorized by the population density of the residential area into four levels, with level 1 as the most urbanized and level 4 as the least urbanized*.

d *New Taiwan (NT) Dollars per month. One NT Dollar equals to 0.03 US Dollar*.

* P < 0.05;

** P < 0.01;

*** *P < 0.001*.

### Influence of mean daily dose for statins on risk of PUD

We further estimated the risk of PUD according to the cumulative defined daily dose (DDD) for each type of statin. As shown in Table 3, statin users were markedly associated with a lower risk of PUD when compared with non-users. In comparison with non-statin users, patients with a cumulative DDD of fluvastatin ≥280 had the lowest risk of PUD (aOR = 0.58, 95% CI = 0.46–0.74), followed by patients with a cumulative DDD of atorvastatin ≥200 (aOR = 0.67, 95% CI = 0.57–0.78), patients with a cumulative DDD of rosuvastatin ≥230 (aOR = 0.71, 95% CI = 0.54–0.93), patients with a cumulative DDD of pravastatin ≥130 (aOR = 0.71, 95% CI = 0.56–0.91), and patients with a cumulative DDD of simvastatin ≥160 (aOR = 0.82, 95% CI = 0.69–0.98). Collectively, these data indicate that the use of statins reduces the risk of PUD, and that this risk was associated with the high cumulative DDD of prescribed statins.

**Table 3:** **Prescribed statins reduce the risk of PUD^**a**^**.

	**Case number/control number**	**Crude odds ratio**	**(95% CI)**	**Adjusted odds ratio^c^**	**(95% CI)**
Non-use of statins	13864/13925	1.00	(Reference)	1.00	(Reference)
Use of statins^b^					
**SIMVASTATIN**					
<160 DDD	789/803	0.99	(0.89, 1.09)	0.81	(0.73, 0.90)^**^
≥160 DDD	272/284	0.96	(0.81, 1.14)	0.82	(0.69, 0.98)^*^
**LOVASTATIN**					
<105 DDD	943/785	1.21	(1.09, 1.33)	1.01	(0.91, 1.11)
≥105 DDD	294/285	1.04	(0.88, 1.22)	0.86	(0.72, 1.02)
**PRAVASTATIN**					
<130 DDD	401/461	0.87	(0.76, 1.00)	0.73	(0.63, 0.84)^***^
≥130 DDD	138/156	0.89	(0.71, 1.12)	0.71	(0.56, 0.91)^***^
**FLUVASTATIN**					
<280 DDD	475/457	1.04	(0.92, 1.19)	0.85	(0.74, 0.98)^*^
≥280 DDD	133/180	0.74	(0.59, 0.93)^**^	0.58	(0.46, 0.74)^***^
**ATORVASTATIN**					
<200 DDD	1124/1189	1.03	(0.95, 1.12)	0.88	(0.81, 0.97)^**^
≥200 DDD	359/441	0.82	(0.71, 0.94)^**^	0.67	(0.57, 0.78)^***^
**ROSUVASTATIN**					
<230 DDD	333/349	0.96	(0.82, 1.12)	0.81	(0.69, 0.95)^**^
≥230 DDD	107/121	0.89	(0.68, 1.15)	0.71	(0.54, 0.93)^**^

a *DDD, defined daily dose; PUD, peptic ulcer diseases; OR, odds ratios*.

b *Cumulative DDD dose divided into 2 segments according to the third quartile*.

c *Adjusted for age, sex, urbanization level, monthly income, aspirin, NSAIDs, tobacco dependency, chronic obstructive pulmonary disease, diabetes, stroke, cirrhosis, coronary artery disease, hypertension, gastroesophageal, reflux disease, gastric polyp, and H. pylori infection*.

* P < 0.05;

** P < 0.01;

*** *P < 0.001*.

To further clarify whether socioeconomic factors were well-balanced between the cases and controls, we carried out a stratified analysis by socioeconomic status. As shown in Table 4, we analyzed the risk of PUD among statin users compared with statin non-users, stratified by sex, urbanization level, monthly income, and smoking related diseases. Compared to the statin non-users, the statin users were significantly associated with lower risk of PUD in the study population for both sexes, with the living in 2^nd^ highest urbanization level, with those of monthly income level between 15,000 and 19,999 and ≥20,000, but without comorbidity with tobacco dependency and stroke.

**Table 4:** **Logistic regression analysis measured the odds ratio for the study group stratified by sex, urbanization level, monthly income, and smoking related diseases^**a**^**.

	**Statin**		
	**Non-PUD group**	**PUD group**	**Adjusted^b^ OR**
**Baseline comorbidities**	***n* (%)**	***n* (%)**	**(95% CI)**
**GENDER**			
Female	1742 (20.3)	1789 (20.9)	0.90 (0.83, 0.98)^*^
Male	1928 (21.4)	1946 (21.6)	0.86 (0.79, 0.93)^**^
**URBANIZATION LEVEL^c^**			
1 (highest)	1365 (21.4)	1226 (22.1)	0.91 (0.82, 1.01)
2	1095 (21.2)	1097 (21.0)	0.86 (0.77, 0.95)^**^
3	561 (20.1)	608 (20.1)	0.87 (0.76, 1.01)
4 (lowest)	649 (19.9)	804 (21.1)	0.85 (0.75, 0.97)
**MONTHLY INCOME (NT$)^d^**			
<15,000	823 (22.3)	794 (22.3)	0.90 (0.79, 1.02)
15,000–19,999	1549 (20.3)	1772 (21.0)	0.89 (0.82, 0.97)^*^
≥20,000	1298 (20.7)	1169 (20.9)	0.84 (0.76, 0.93)^**^
**SMOKING RELATED DISEASES**			
Tobacco Dependency			
No	3607 (20.8)	3649 (21.2)	0.88 (0.83, 0.93)^***^
Yes	63 (27.6)	86 (24.5)	0.72 (0.46, 1.13)
**COPD**			
No	3286 (20.0)	3171 (20.2)	0.88 (0.83, 0.94)^***^
Yes	384 (33.9)	564 (30.1)	0.79 (0.67, 0.94)^*^
**STROKE**			
No	2967 (18.5)	2960 (18.7)	0.87 (0.81, 0.92)^***^
Yes	703 (46.0)	775 (43.4)	0.89 (0.77, 1.03)
**CAD**			
No	2580 (16.9)	2401 (16.7)	0.89 (0.83, 0.95)^*^
Yes	1090 (47.0)	1334 (41.7)	0.82 (0.73, 0.92)^***^

a *CAD, coronary artery disease; COPD, chronic obstructive pulmonary disease; OR, odds ratios; PUD, peptic ulcer diseases*.

b *Mutually adjusted model age, sex, urbanization level, monthly income, aspirin, NSAIDs, tobacco dependency, COPD, diabetes, stroke, cirrhosis, CAD, hypertension, gastroesophageal reflux disease, gastric polyp, and H. pylori infection simultaneously*.

c *The urbanization level was categorized by the population density of the residential area into four levels, with level 1 as the most urbanized and level 4 as the least urbanized*.

d *New Taiwan (NT) Dollars per month. One NT Dollar equals to 0.03 US Dollar*.

* P < 0.05;

** P < 0.01;

*** *P < 0.001*.

## Discussion

The results of the present study showed that after adjusting for risk factors including age, sex, urbanization level, monthly income, aspirin, NSAIDs, and the major comorbidities (aOR = 0.87, CI = 0.82–0.93, *P* < 0.001), patients who were prescribed statins associated with a significantly lower risk of PUD than those who were not. Overall, the results of this study suggest that statin use significantly reduces the risk of PUD. In addition, these findings are consistent with studies previously conducted in other countries (Heeba et al., 2009; Nseir et al., 2010, 2012).

Statins prescribed in Taiwan include lovastatin, simvastatin, pravastatin, fluvastatin, atorvastatin, and rosuvastatin (Tsan et al., 2012). These statins are commonly prescribed as cholesterol-lowering agents in patients with hyperlipidemia and are considered to exert anti-inflammatory activity, as indicated by reduced levels of the inflammatory marker C-reactive protein in patients who are prescribed statins (Ridker et al., 2008). Existing clinical evidence suggests that statins reduce the incidence of coronary disease (Nissen et al., 2005; Ridker et al., 2005), rheumatoid arthritis (Chodick et al., 2010), Alzheimer's disease (Di Paolo and Kim, 2011), chronic obstructive pulmonary disease (Cao et al., 2015), and gastric cancer (Lin et al., 2016). Most importantly, statins have been reported to protect against bacterial infectious diseases (Jerwood and Cohen, 2008; Nseir et al., 2013; Skerry et al., 2014; Lin et al., 2016). The combination treatments including triple therapies and statins facilitated *H. pylori* clearance and attenuated ulcer development (Tariq et al., 2007; Yamato et al., 2007; Nseir et al., 2012). In addition, our recent study revealed that statin treatment reduced *H. pylori* burden in macrophages to alleviate *H*. *pylori*-associated pathogenesis (Liao et al., 2017). However, the molecular mechanisms underlying the effects of statins on the risk of such diseases remain to be investigated.

Although our previous study reported the protective effects of statins against peptic ulcer (Feng et al., 2015), several limitations and confounding factors exist. In the current study, we further analyzed the potential confounding medications including aspirin and NSAIDs. In addition, various statins that are commonly used in Taiwan (i.e., simvastatin, lovastatin, pravastatin, fluvastatin, atorvastatin, and rosuvastatin) were classified and the cumulative DDD of each type of statin was analyzed. Moreover, to further clarify whether socioeconomic factors were well balanced between the cases and controls, we performed a stratified analysis based on socioeconomic status such as urbanization level and monthly income. Therefore, the findings presented in the current manuscript have greatly improved with further advancement of knowledge.

Although the strength of the analyses of population-based data that possess highly representative of the general population, certain confounders and limitations emerged from our results should be considered. First, the data we collected from NHIRD contains only the disease and treatment received of patients (Cheng et al., 2011). We did not obtain the detailed information such as: occupation, alcoholic, exercise, body mass index, smoking habit, and environmental exposure, which are all potential confounding variables. Second, the evidence derived from a population-based case-control study is generally lower in statistical quality than that from randomized trials because of potential biases related to adjustments for confounding variables (Hsu C. C. et al., 2015). Third, all data in the NHIRD are anonymous, the relevant clinical variables, such as imaging results, pathology findings, and serum laboratory data were unavailable for our study cases (Chen et al., 2015). Finally, changes in statins habits over time are inevitable and may result in exposure misclassification and attenuation of the association between statin intake and PUD. Moreover, the inconsistent results might in part be due to measurement error in the assessment of different statins, diet including inaccurate recall, changes in dose over time. However, Taiwan launched a NHI program operated by a single-payer, the government, in 1995. All insurance claims are scrutinized by medical reimbursement specialists and subject to peer review. Therefore, the data regarding the diagnoses for patients with PUD and statin prescriptions were reliable.

In the present study, a population-based case-control study showed that patients who were prescribed statins had a significantly reduced incidence of PUD. Our study using a nationwide population analysis indicate that statins may be used to protect against PUD.

## Author contributions

Conceived and designed the experiments: CK and CHL Performed the experiments and analyzed the data: CJL, WL, YC, HL, MH, and CF. Wrote the manuscript: CLL, YL, MK, CJL, and WL Revised the manuscript: CJL, WL, YC, HL, CLL, CK, and CHL. Reviewed the final version of this manuscript: all authors.

## Funding

This study is supported in part by Taiwan Ministry of Health and Welfare Clinical Trial and Research Center of Excellence (MOHW106-TDU-B-212-113004), China Medical University and Hospital (CMU102-BC-2, DMR-103-018, and DMR-103-020), Academia Sinica Taiwan Biobank Stroke Biosignature Project (BM10501010037), NRPB Stroke Clinical Trial Consortium (MOST105-2325-B-039-003), Tseng-Lien Lin Foundation, Taichung, Taiwan, Taiwan Brain Disease Foundation, Taipei, Taiwan, Ministry of Science and Technology (104-2320-B-182-040 and 105-2313-B-182-001), Chang Gung Memorial Hospital (CMRPD1F0011-3, CMRPD1F0431-3, and BMRPE90), Tomorrow Medical Foundation, and Katsuzo and Kiyo Aoshima Memorial Funds, Japan. The funders had no role in study design, data collection and analysis, decision to publish, or preparation of the manuscript. No additional external funding received for this study.

### Conflict of interest statement

The authors declare that the research was conducted in the absence of any commercial or financial relationships that could be construed as a potential conflict of interest.
